# Biological Well-Being during the “Economic Miracle” in Spain: Height, Weight and Body Mass Index of Conscripts in the City of Madrid, 1955–1974

**DOI:** 10.3390/ijerph182412885

**Published:** 2021-12-07

**Authors:** Elena Sánchez-García, José-Miguel Martinez-Carrión, Jose Manuel Terán, Carlos Varea

**Affiliations:** 1 Department of Biology, Faculty of Sciences, Madrid Autonomous University, 28049 Madrid, Spain; josemanuel.teran@alumni.uam.es (J.M.T.); carlos.varea@uam.es (C.V.); 2 Department of Applied Economics, Faculty of Economics and Business, Murcia University, 30100 Murcia, Spain; jcarrion@um.es

**Keywords:** anthropometry, secular trends, inequality, body mass index, conscripts, Spain

## Abstract

Typifying historical populations using anthropometric indicators such as height, BMI and weight allows for an analysis of the prevalence of obesity and malnutrition. This study evaluates secular changes in height, weight and body mass for men cohorts at 21 years old, born between 1934 and 1954 who were called up between 1955 and 1974, in the city of Madrid, Spain. In this study we prove the hypothesis that anthropometric variables increase thanks to improvement in diet and significant investments in hygiene and health infrastructure during the 1960s. The results of our analysis show a positive secular change in the trends for height (an increase of 4.67 cm), weight (6.400 kg) and BMI (0.90 Kg/m^2^), the result of a recovery in standards of living following the war and the autarchy of the 1940s. We also observed a slight trend towards obesity and a reduction in underweight categories at the end of the period is also observed. In conclusion, the secular trends of anthropometric variables in the city of Madrid reflect the recovery of living standards after the deterioration of the nutritional status suffered during the Spanish Civil War (1936–1939) and the deprivation of the autarchic period.

## 1. Introduction

The human life cycle is characterised by a long period of growth which provides great biological plasticity, allowing us to adapt to changing environmental situations [1]. The speed of growth changes over the different stages of the life cycle, being at its highest in the foetal stage, childhood and adolescence [2]. During these critical stages, a negative energy balance due to lack of food, physical effort, infections or stressful situations affects development irreversibly [3]. This biological plasticity can be seen in the so-called secular trends, somatic and physiological modifications in successive cohorts due to environmental change, and therefore negative, positive and reversible [4].

The study of differences in adult height allows us to evaluate both social inequality and living conditions for individuals from the same cohort, such as time trends linked to socio-economic changes in the same population [5,6]. Hatton and Bray [7] have described an average increase in height of 11 cm in 15 western European countries in cohorts born between the mid-19th to the second half of the 20th centuries, that is, one centimeter per decade. This increase was intense and came sooner in countries in northern and central Europe, with the fastest growth in the periods 1911–1915 and 1951–1955, whereas in southern Europe it was in the periods 1951–1955 and 1976–1980 [7,8].The strong increase in height and in other biological variables in developed countries has been linked to socioeconomic growth, mainly to improvements in nutrition, income, public health and education—the so-called Technophysio evolution [9,10].

In turn, the study of body fat as an indicator of obesity and a predictor of cardiovascular risk in adults has been taken on by Biological Anthropometry and Epidemiology using parameters and variables linked to the accumulation of visceral fat [11,12]. One problem with such studies in historical populations is the lack of data for variables such as waist circumference and the waist-hip relation among others. Therefore, body mass index (BMI = weight in kg/height in m^2^) has the advantage of being easy to apply to the available data for height and weight and of having a high correlation with other indicators of adiposity [13]. The use of BMI in historical populations has been frequent for examining Net nutrition and health, since Hans Waaler made clear the connection which exists between high BMI and the risk of premature death [14,15,16,17,18]. However, BMI has also been a very controversial indicator given that it does not take into account the different aspects of body weight, which vary greatly among the population [13]. In spite of this, high BMI levels have been linked to the risk of higher systolic and diastolic arterial pressure, higher lipoprotein cholesterol of very low, low and high density, triglycerides, and high insulin levels [19]. A high BMI has also been linked to illnesses (type II diabetes, hypertension, coronary disease and other cardiovascular diseases [20,21]) and the inflammation of the adipose tissue, by the accumulation of adipokines and macrophages in fat tissues increasing the production of proinflammatory mediators [22,23] On the other hand, low BMI levels are linked to anemia, collapse of the immune system, osteoporosis, nutritional deficit, menstrual irregularities and a reduction of cognitive faculties [24,25]. Thus, the first cut off points regarding degrees of thinness (BMI < 18.5) were established via measurements of the basal metabolic rate [26] and excess weight (BMI > 24.9) starting with the link between BMI and mortality [27,28]. Currently, the categories for BMI established by the World Health Organization (WHO) [29] are: low weight, BMI < 18.50; normal weight, BMI = 18.50–24.90; pre-obesity, BMI = 25.00–29.90; and type I obesity, BMI = 30.00–34.90.

Together, height, weight and BMI have been widely used for the study of historic populations. Height allows us to address the analysis of living conditions during the first stages of development, while weight provides information about the nutritional state at the moment of measuring. For its part, BMI allows for a joint evaluation of both processes, establishing the prevalence of malnutrition [19,30]. Typifying adiposity in populations has gained special importance due to the current obesity epidemic [31,32]. Since the 1960s, we have seen a drop in the prevalence of low weight and a rise in obesity in high income nations, mainly the United States [33,34,35] but also the whole of Europe [36,37,38,39] and Asia [40,41]. The magnitude of these changes has varied significantly in different parts of the world, affecting changes in the distribution of BMI [42,43,44]. This increase in the prevalence of child and adult obesity, which started in most high-income nations in the 1970s, spread to low and middle-income nations in the 1980s and 90s [45], mainly in rich, urban areas. The rapid demographic growth of many cities due to the massive migratory flow and faster urbanisation, which modified diet and physical activity, and affected the environment, were determinants for the increased prevalence of obesity [46].

In sum, the variability in height and weight (and, accordingly, in BMI) through time and among populations is the expression of the complex interrelation between genes and external environment conditions. A certain—debated—percentage of the phenotypic variation in anthropometric variables within a given population is explained by additive genes, but the sustained and slow increase in adult height between the mid-19th to the second half of the 20th centuries in developed countries adequately fits the equally slow pace of socioeconomics changes affecting through epigenetic growth regulators over successive generations, while secular trends in weigh express the direct and immediate impact of changes in nutrition and lifestyles throughout the individual life course. As a result, weight is not as reliable an indicator of healthy growth [19].

As far as we know, this is the first study of annual series of height and weight which evaluates secular trends in Spain in BMI before the modern obesity epidemic and following the prolonged period of economic isolation (autarchy) [47]. We have at our disposal important analyses of trends in height in Spain which include regional evidence from the 20th century, and in particular the cohorts from 1950 to 1980 [7,8,48,49,50]. However, we know very little about trends in weight and BMI before 1980. This research addresses the anthropometry and somatology of the male population of Madrid (Spain) between 1955 and 1974 using height, weight and BMI. Our previous studies of height in the city of Madrid show a significant global increase for the cohorts born in the first half of the 20th century, albeit with noticeable socioeconomic differences depending on district of residence [51,52,53]. In the country as a whole, this period is characterised by the change from the tough economic and social conditions of the autarchy to the rapid economic growth in the 1960s and early 70s. After the end of the Spanish Civil War (1936–1939) and General Franco’s victory, a regime of economic isolation (autarchy) was imposed which led to a fall in agricultural and industrial production, and a slowing down of economic development [54]. During the first decade of the post-war the country went through a deep economic depression and poverty, which reduced levels of material well-being as has never been seen in contemporary history. Due to this difficult socio-economic situation, large-scale migration from the countryside to the cities arose, which created a vast unskilled workforce of paid employees in the urban world, a situation which intensified during the 1950s [55]. In the case of Madrid, the migrant population set up settlements on the outskirts of the capital in areas with poor hygiene and sanitation [56,57] where poverty, slums and segregation abounded [54]. As from 1951, both the change in Franco’s economic policies and the end of international isolation took the regime into the exterior market boosted by industrialisation and an increase of capital investment which allowed the country to recover economic development [58,59]. In Madrid, massive construction projects arose to build new towns, townships and temporary settlements to improve the living conditions of the people who lived scattered around the periphery in bad housing [60].

**Figure 1:**
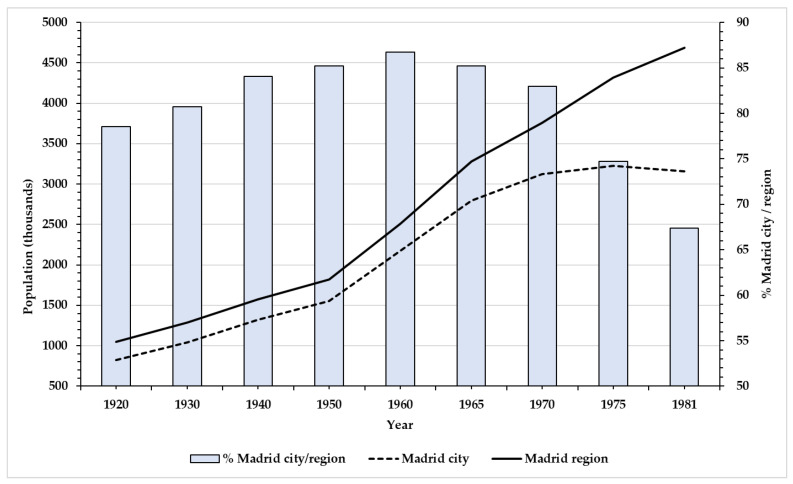
Population of the city of Madrid and its region, 1920–1981 (Source: *Instituto Nacional de Estadística,* INE) Population of Madrid since 1920.

With the *Plan de Establización y Liberalización Económica* (Stabilisation and Liberalisation Plan) of 1959 and especially the 1960s and early 1970s the so-called “Spanish economic miracle” [61] came about, characterised by high levels of economic growth, industrialisation and rapid urbanisation [62,63,64]. In Madrid the “economic miracle” saw one of its major achievements, boosting industrialisation and rapid urbanisation [62,65]. The city underwent a huge demographic growth, fed by immigration, and became the most populated municipality in Spain [66]. The city trebled its demographic size between 1940 and 1970 (Figure 1), which meant successive planning for land management and more housing [67]. In this time frame, our purpose in to contrast the hypothesis that anthropometric variables increase during the 1960s due to improvement in diet and significant investments in hygiene and health infrastructure.

## 2. Materials and Methods

### 2.1. Data Source

**Figure 2:**
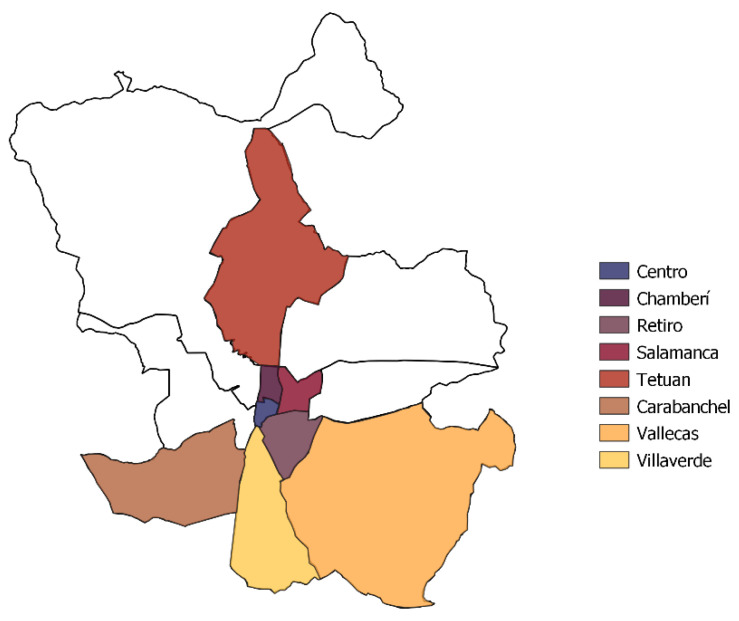
Map of Madrid and the analysed districts.

This study analyses secular trends in height, weight and Body Mass Index (BMI) in conscripts called up in Madrid between 1955 and 1974, a crucial period for the modernisation of Spain. The data analysed comes from what are known as the *Libros Filiadores de Madrid* (LFM), a historical source kept in the Guadalajara General Military Archive (*Archivo General Militar de Guadalajara*, AGMG) and unpublished [51,52,53]. The LFM include information about the conscripts, their home address, date of birth, anthropometric variables, allegations made, and extension requests granted, among others. It was drawn by year and by district with information for between 400 and 600 individuals, representing between 30 and 40% of the total male population of the city, including those who were finally rejected for military service, reaching a total of 42,664 individual registers of which 76.6% (32,692) offer anthropometric data. The conscripts were called up aged 21 between 1955 and 1970 (cohorts from 1934 to 1949) and aged 20 as from 1970 to 1974 (cohort of 1950 to 1954). The population analysed corresponds to residents in eight districts of the capital (Salamanca, Villaverde, Tetuán, Centro, Vallecas, Latina, Chamberí and Retiro: Figure 2) chosen because of their contiguity and social character [53]. These eight districts have been grouped in two categories: lower class (Tetuán, Villavede, Latina and Vallecas) and middle and upper classes (Centro, Salamanca, Retiro and Chamberí) due their contrasted socio-economic conditions [51,52].

### 2.2. Statistical Analysis

First of all, we evaluate time trends in the average values for height, weight and BMI throughout the period under study using quadratic regressions. Quadratic regressions are used because they have shown better fits in previous studies [51]. For the BMI categories χ^2^ analysis have been used because of its robustness with respect to distribution of the data [68].

Secondly, temporary series for height, weight and BMI in Madrid were compared to the reference values set by the WHO (2007) at the age of 19 [69] (height: 176.50; weight: 69.16; BMI: 22.20) and with the data for Spanish male population [70] at 18 years old (height: 175.97; weight: 70.20; BMI: 22.60).To this end, we have established the Z-score values for BMI for age (BAZ), Z-score values for height for age (HAZ) and Z-score values for weight for age (WAZ) using WHO values at the age of 19.

HAZ below −2.00 SD is considered an indicator of retarded growth. In the case of WAZ values below <−2.00 SD and <−3.00 SD indicate thinness and severe thinness, and values for BAZ over 1.00 SD indicate overweight and above 2.00 SD obesity [28,71,72].

Analyses were performed by SPSS (version 22, IBM Corporation, Armonk, NY, USA) and RStudio (version 3.5.1, RStudio, Inc., Boston, MA, USA) statistic programs.

## 3. Results

**Figure 3:**
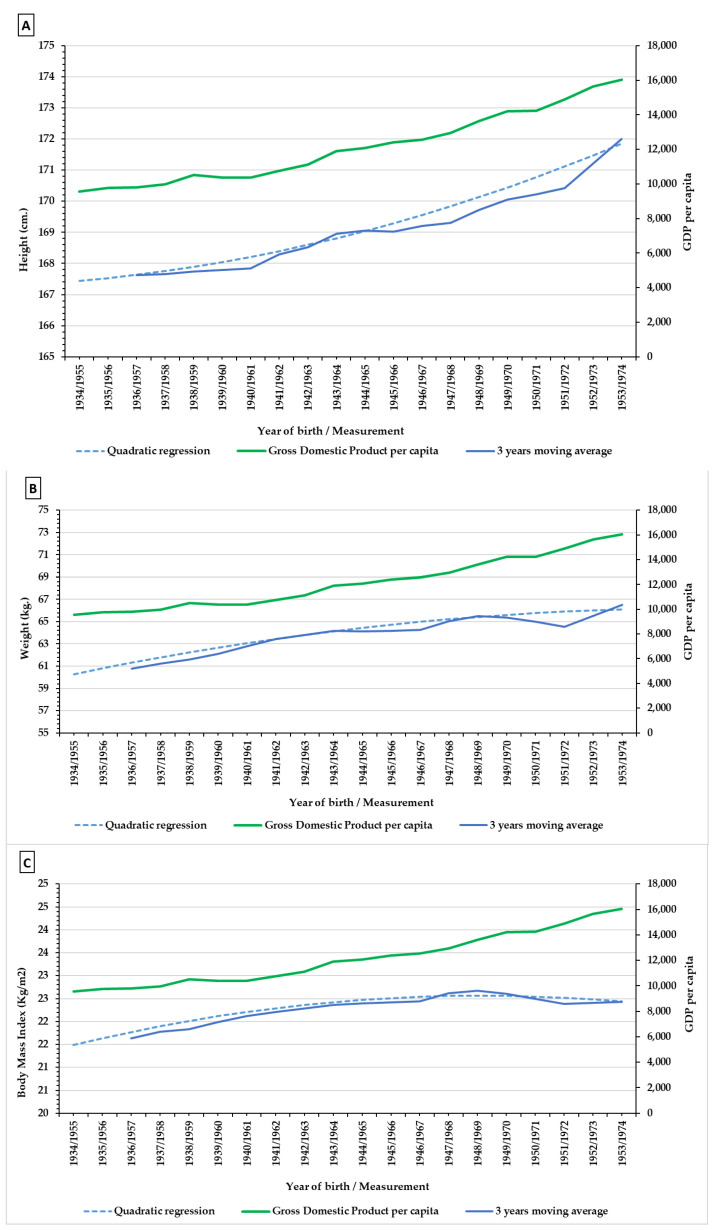
Secular trends and mobile means of 3 years in anthropometric variables (Blue), height (**A**), weight (**B**) and body mass index (**C**) during the period of 1955–1974. In comparison with GDP (Green) (R^2^ height = 0.92; R^2^ weight = 0.92; R^2^ BMI = 0.89). (Source, AGMG).

**Figure 4:**
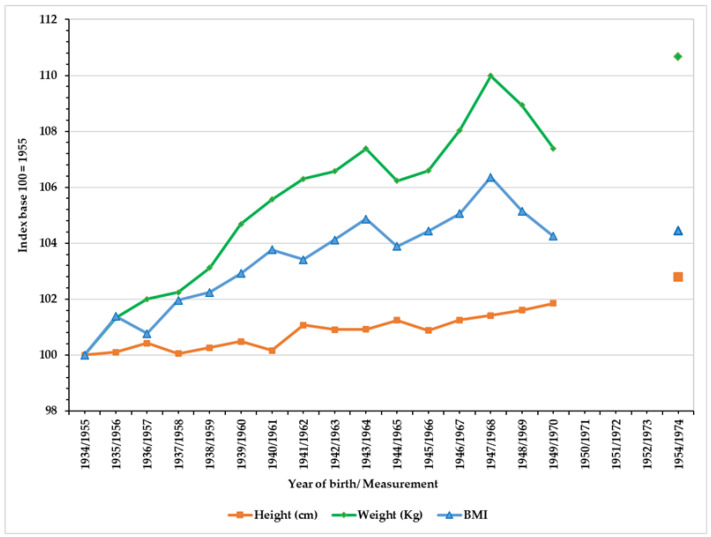
Increases in anthropometric variables in the city of Madrid, 1955–1974. Cohorts 1934–1954 (base 100 = 1955). (Source, AGMG).

Figure 3 (see Table A1 in the Appendix A) shows secular trends for the average values of the anthropometric variables analysed, and the Gross National Product GNP per capita for the region of Madrid. The three variables show a significant temporary increase (R^2^ height = 0.92; *p* value < 0; R^2^ weight = 0.92; *p* value < 0; R^2^ BMI = 0.89; *p* value < 0). Height increased from 167.33 cm in 1955 to 172.00 cm in 1974, 4.67 cm. Weight increased 6.400 kg in the period in question, from 60.10 to 66.51 kg, with a tendency slowdown from the middle 1960s. Finally, BMI increased by 0.90 Kg/m^2^, from 21.48 to 22.43 Kg/m^2^, and shows the temporal pattern of the sustained increase in height and the slowdown in the increase in weight from the middle 1960s, levelling out around values of 22.50 Kg/m^2^. Figure 4 allows us to appreciate the intensity of the percentage increase in the three variables described, taking 100 as the value for the first year in the series.

Table 1 shows temporal change in the distribution of BMI (X^2^ = 507.97; *p* value = 0.00 and gl = 84). We can appreciate a sustained increase in the prevalence of pre-obesity (which doubles) and a clear drop in the low weight category (which reduces to half) while there is a slight decrease in the percentage for normal weight, which is the predominant category for the whole period.

**Figure 5:**
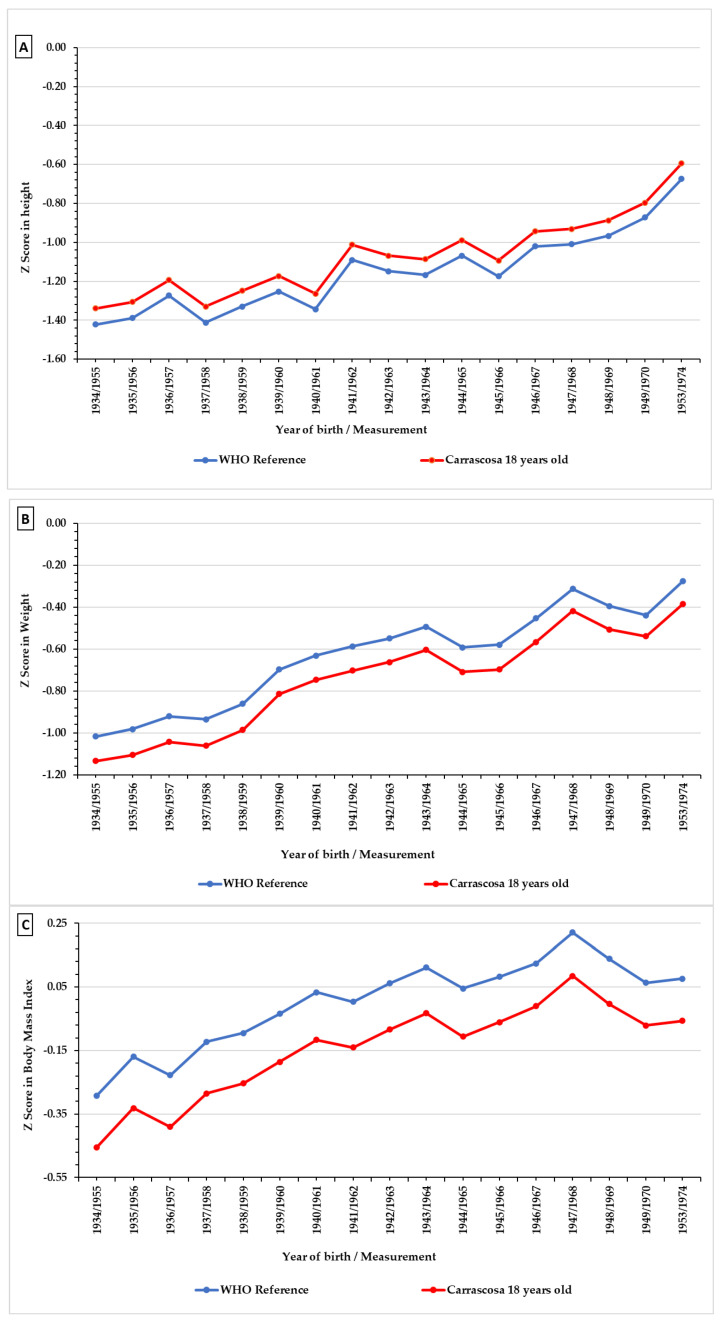
Z-score by year from 1955 to 1974 in height (**A**), weight (**B**) and BMI (**C**). (Source, AGMG).

The Z-score results for height show (Figure 5A) values of around −0.5 and −1.5 standard deviations from the WHO reference values [69] and Carrascosa et al. [70]. In both cases the sample is under the 1 deviation typical for the reference values until the second half of the 1960s. In the case of weight, the differences (Figure 5B) between the reference values and those in the sample are lower. At the beginning of the simple the values are of around −1 standard deviation from international and Spanish values. However, these differences reduce sharply varying between −0.2 and −0.4 standard deviations. Relative decline in Z-score weight was higher than in height, particularly in the later years, although it is also more affected by the increases in the 1960s.In the case of BMI (Figure 5C) values are in the range of −0.5 standard deviations from the reference values for the years before 1960, during the following years they are around 0.25 standard deviation. Values for BMI for the 1961 conscription reach those established by the WHO, and from that year onward exceed the percentile values set by the WHO. As for the data presented by Carrascosa et al. [70] this equivalence comes about later, in 1967, and as with the previous case exceeds the same in following years albeit with fluctuations.

## 4. Discussion

Since the end of the 19th century, the physical stature of Europeans experienced the greatest growth in its history. The strong increase in the size of the bodies was the response to the fabulous economic growth and expansion of the Welfare State, which had its greatest expression after World War II. In Western Europe and the rest of the high-income countries, the years 1950 to 1973 were of general prosperity [73]. In this period, the indicators of the standard of living experienced strong growth: per capita income, income, life expectancy, consumption, education, and there was a sharp decline in infant mortality.

In Spain this prosperity became noticeable after the 1959 Stabilisation and Liberalisation Plan. With this change of direction in economic policy, the 1960s saw an acceleration in the process of industrialisation and urbanisation, and there was progress in mass education and public healthcare [74]. The economic reforms were decisive for the liberalisation of the Spanish economy and its integration into the international market, industrial development and economic growth [75,76]. Despite being a state with weak institutions, the foundations were created in the 1950s and 1960s for the country to escape the poverty trap and enter the developed world of high-income countries [77].

Madrid became the powerhouse of the Spanish economy together with Catalonia and the Basque Country. The data for per capita income available since 1955 put Madrid at the top of the Spanish economic regions in the middle 1970s. From the early 1960s to the middle 1970s, Madrid’s regional economy grew considerably, and was considered one of the richest areas of Spain [63]. It was the first to hold the greatest number of people employed in the service sector, and the second in industry and construction. As from the early 60s Madrid became a large industrial centre and the capital of the service sector [76]. In addition, as of 1964, it boasted the highest average salary in Spain and, in 1975 the highest per capita wealth in the country [78]. This economic and demographic expansion in Madrid was the result of two processes: the epidemiological transition which led to a drop in mortality rates as a result of improved sanitation [79] and the contribution of migrants linked to the rise of industry and the growth of the service sector [54].

The improvements to health and living conditions from 1950 to 1970–1980 were significant in the country as a whole and particularly in Madrid, the capital. Life expectancy at birth for Spaniards increased by 13.4 years between 1950 and 1980, the increase amongst women being notable. For men, it went from 59.8 years in 1950 to an average of 72.5 years in 1980 [80]. Spain registered one of the major advances in Western Europe [81]. The “conquest of health” as followed by a drop in the general mortality rate in the mid-century decades, something that was intensifying in the first half of the 20th century. The infant mortality rate, a good indicator of standards of living, saw important progress. In Spain as a whole, it went from 69.8 per 1000 in 1950 to 28 per 1000 in 1970. Its evolution in the city of Madrid contrasts with that in the province of Madrid, where results for the same period were worse. Around 1950, the infant mortality rate in the province was 88.6 per 1000 compared to 56.2 per 1000 in the capital. This difference of over 30 points explains the strong attraction which, in terms of health, the capital held for the rest of the province and the country as a whole. The advances in human wellbeing in the province and capital of Madrid were notable during the decades of the economic ‘miracle’, following improvements to amenities and infrastructure in many places, mainly urban but also in rural areas, particularly those concerned with water supply and drainage. In 1970, the provincial infant mortality rate was 36 per 1000 whereas in the capital it reached only 24.80 per 1000 [82]. Previous studies shows that differences in height between people from the city of Madrid contrasts with people from the province of Madrid. These differences are about 0.11 cm in 1956 (166.94 cm in the city and 166.83 cm in the province) and increase to 0.44 cm in 1974. The maximum differences are recorded in 1960 (1.55 cm) and are declined in following decades [49]. The urban-rural differences were still important, but the gap between the two worlds had been reduced, due to progress in nutrition and a diet which was more suited to physiological requirements, the consumption of animal proteins, particularly milk products, being the most important [83].

The study of secular trends in anthropometric indicators provides valuable information about trends in health, nutritional state and levels of biological life during the growth of the population during a specific period [5,6]. In the case of Madrid, research into the height of conscripts shows a sustained increase in height throughout the 20th century and brings to light the enormous spatial segregation in the city depending on socio-economic levels [51,52]. This study focuses on the period from 1955 to 1974. It is characterised by modernisation, the industrialisation of Spain and huge progress in mass education and public healthcare [74,75,76]. This period documents the recovery of the battered levels of biological life after the previous two decades of autarchy.

During this period of growth and transformation in the Spanish capital we can see a positive secular trend in the three anthropometric variables which reflects an improvement in living conditions (Figures and Tables). In only two decades, the population of Madrid had one of the biggest increases in height documented in its history, 4.70 cm, an average higher than the average for Spain which for the same period was 3.70 cm [49]. During these decades, Spain figured among the countries with the highest rates of increase in height, and so the figures for Madrid reflect the intensity of the socio-economic transformation [5,7,8]. Similarly, weight increased by 6.40 kg from the value 60.10 in 1955 (born in 1934) to 66.50 kg in 1974 (born in 1954). This increase is discreet and less than that seen for height (Figure 4). However, it indicates the social and economic recovery which came about after the autarchy. As is the case with height, weight increases more intensely in the later years of the period in question: between 1963 and 1974 it increased by 2.62 kg, despite a noticeable decrease in the averages in the late 1960s. As for BMI, the increase was of 0.95 points, from a BMI of 21.48 in 1955 (born in 1934) to a BMI of 22.43 in 1974 (born in 1954). This increase reaches a maximum between 1965 and 1968 with a rise of 0.53.

The conscripts during the first years of our temporal series were born during the Civil War and their childhood was during the war and the long post-war period. “The years of hunger”-as the severe malnutrition period from 1941 to 1946 are called-may have been decisive for final height. The 1940s was a decade of black market, deprivation and shortage of basic foodstuffs, brutal inflation, ration books, restricted water provisioning and power cuts, as well as the collapse of real salaries [84,85], the worsening of working conditions and the resurgence of infectious diseases such as typhus and tuberculosis [81]. This explains that the height adults in Madrid and in Spain as whole were among the lowest in Europe in the early 1950s.Rationing ended in 1952 and the following years saw lukewarm reforms leading to a transition period which did not bear fruit until 1959 Stabilization and Liberalization Plan. It is from then that the autarchic model changes definitively to an occidental one based on the freeing up of the markets. The transition from the autarchy of the first period of the Franco dictatorship to the “developmentalism” of the later period had enormous repercussions on the living conditions of the Madrid population.

The decade of the 60s represents a notable change in the lifestyles of the Madrid population, mainly urban. The city registered a real urban, social and economic transformation. In just a decade, it grew from two to three million inhabitants and became a cosmopolitan city. The composition of private consumption changed dramatically between the mid-1950s and the mid-1970s. There was a greater propensity to spend on durable goods, including culture and leisure, consequence of the increase in real family income and per capita income. New food markets were built that tended to meet food demands due to demographic expansion and urban growth. The pressure of the vicinity movement in the popular neighbourhoods and the suburbs began to spread the need for political changes, rights and freedoms, which would crystallize with the end of the Franco regime in 1975 [86].

Madrid boasted one of the fastest rates of economic growth in Spain. The increase in incomes and earnings improved diet and eating habits which led to an increase in calorie intake and an improved quality of nutrients (mainly milk and meat) [87,88]. Although meat consumption is still poorly studied in Spain, data reveals a faster growth in its consumption than in dairy products among 1955–1975. During the period of 1960–1968, the meat consumption reaches a mean of 83.9 gr per capita and day. It became the main contributor on caloric and lipid ingestion in Spain [89]. In global terms, the evolution of meat consumption per capita and year were doubled from 1965 to 1975 [90]. The 196465 Family Budget Survey (*Encuesta de Presupuestos Familiares*) shows that the average consumption of nutrients per capita and day in the city of Madrid was higher than that of urban Spain in beef and pork, fresh fish, eggs, fresh fruits and, above all, all in fresh milk. Diet composition data reflect a marked improvement in its quality in the mid-1960s. Data from a survey of nutrition among Spaniards in1970 show that the people of Madrid had their nutritional needs covered (see Table A2 in the Appendix B) [91]. Added to this, there was an improvement in maternal and infant health, education and living infrastructure. Already in 1962, the data show a first significant rise in the height of the conscripts. The increase in height, weight and BMI in the 1960s and until 1974 reflects the environmental improvements of their adolescence and allowed for the recovery of their potential growth which they had lost due to the deterioration of their nutritional state and health in their early infancy [92].

In Western European countries in cohorts born between the mid-19th to the second half of the 20th centuries increased one centimetre per decade [7]. This increase was intense and came sooner in countries in northern and central Europe, whereas in southern Europe it happens in the periods 1951–1955 and 1976–1980 [93]. In Greece for example, the period from 1951 to 1965 increased significantly the stature. Previous decades were characterised by war and famine. In fact, the decline of height in the period of 1935 to 1945 was so dramatic that in comparison with Madrid statures, Greek conscripts were 2.14 cm smaller than those of Madrid [94]. In other southern countries such as Italy the war-related deprivation seems not to have affected adult height. On the contrary, the highest rates of growth were recorded for the generations born in the 1940s and 1950s maybe because the World War II were immediately followed by a long cycle of economic growth, during which children were nearly able to reach their physical potential for growth [95].

A conclusive evaluation of the recovery of growth could be inferred from the average Z Scores of the conscripts with reference to their modern Spanish equivalents and to the healthy populations as established by the WHO (Figure 5). On comparing the values for height, weight, and BMI for the population of Madrid with the international and national standards we can see that retarded growth corrected itself. The Z Scores reduce considerably towards 1974 due to the sudden rise registered in the height of young men in the 1960s, and the same trend can be seen in weight, although in the latter case the reduction in differences is less constant. It is important to highlight the evolution of BMI, its values are above national and international ones in the 1960s, which reflects the environmental improvements.

These results can be compared with the work of Salvatore (2020) [96] in Buenos Aires. There are no similar studies of malnutrition in cities, so the case of Buenos Aires is a good reference. In his study, Salvatore analyses data between 1850 and 1950 from different sources. In this period industrialization and demographic increase makes Buenos Aires a modern big city that could be compared with Madrid. The results show an important de-cline in malnutrition percentages since 7.29% in 1900 to 2.72% in 1940. In the city of Madrid, our analysis does not show malnutrition. Z Scores are maintaining under the 1 deviation typical for the reference values until the second half of the 1960s.

As regards BMI, during the first decade of the period analysed (1955–1959) the categories for healthy weight predominate, although there is a significant percentage of the population with low weight (5%) and obesity (8%). This distribution varies in the later periods (1960–1969) where we can see a gradual trend in the values towards overweight (14.9% of the population 1965–1969). The results show that the improvement in living conditions during the 1960s caused a change in the eating habits and lifestyle of the population, setting a trend which accelerated in the 1980s [79]. This change in eating habits started in the 1950s, when the consumption of meat, fish, dairy products and fruit increased [97]. Milk consumption increased significantly in the 1960s, thanks to the increase in the supply of dairy plants and the improvement in commercial distribution [92,98]. This increase in protein intake and specifically milk is linked to improvements in development conditions and is particularly reflected in height [99]. In this context, the following gain importance: the consumption of dairy products and the reduction of illnesses thanks to the general use of pasteurisation and sterilisation of milk, or the appearance of condensed and dried milk [100]. Nowadays, obesity affects in Spain 22.8% of the men and 20.5% of women, due to differences in physical activity and diet [47].

As is well known, data on temporal series on anthropometric variables are restricted to men, as women was excluded from the compulsory military service. Beside this limitation, in our study data on weight is only available from 1955 onwards, while data on height and other anthropometric variables are accessible since 1936.

## 5. Conclusions

The results of this analysis show a positive secular trend in the trends for height, weight and BMI for the period 1955–1974 in the city of Madrid. The evolution of anthropometric values reflects the recovery of living standards after the deterioration of the nutritional status suffered during the armed conflict (1936–1939) and the deprivation of the autarkic period. The period that followed 1959 was characterised by economic reforms that promoted liberalisation and integration of the Spanish economy into the international markets, industrial development, and economic growth [71,72]. Previous studies showed that the Civil War and subsequent years of famine affected the growth of conscripts at a critical stage of their development, such as childhood and adolescence, including those living in Madrid between 1936 and 1974 [50,51]. Data from this study reveal the improvements that took place since the 1960s with an increase in height and weight, and an incipient tendency towards overweight categories.

The population of Madrid was able to benefit from the important investments made in the infrastructure of hygiene and healthcare because of being the capital. The situation also favoured an improvement in per capita income and diet. The increase in calorie intake and improved nutrients (from animal proteins: meat and milk, mainly) led to an increase in height and other anthropometric indicators such as weight and BMI. Thus, they approached the anthropometric standards of modern European populations. Future research focused more on residential and social differentiation might cast light on whether this dramatic increase in height, weight and BMI had unequal effects depending on neighbourhood and social class during this period of maximum economic growth in Spain.

## Figures and Tables

**Table 1:** Relative distribution of the different categories of BMI in the city of Madrid from 1955 to 1974. Low weight BMI < 18.50; normal weight, BMI = 18.50–24.90; pre-obesity, BMI = 25.00–29.90; and obesity type I, BMI = 30.0–34.90) (Source: AGMG).

Year of Birth/ Measurement	Low Weight	Normal Weight	Preobesity	Obesity Type I	*N* Total
	% (*N*)	% (*N*)	% (*N*)	% (*N*)	
1934/1955	6.39 (61)	85.48 (842)	7.21 (71)	0.91 (9)	985
1935/1956	5.73 (91)	84.26 (1338)	9.26 (147)	0.76 (12)	1588
1936/1957	6.38 (140)	84.59 (1856)	8.16 (179)	0.87 (19)	2194
1937/1958	3.50 (69)	86.26 (1702)	9.07 (179)	1.17 (23)	1973
1938/1959	4.46 (87)	84.89 (1657)	9.58 (187)	1.08 (21)	1952
1939/1960	3.70 (60)	83.36 (1343)	11.73 (189)	1.18 (19)	1611
1940/1961	3.41 (52)	82.45 (1259)	12.64 (193)	1.51 (23)	1527
1941/1962	4.55 (58)	81.96 (1045)	12.00 (153)	1.49 (19)	1275
1942/1963	3.56 (67)	81.46 (1533)	13.12 (247)	1.86 (35)	1882
1943/1964	3.49 (63)	79.83 (1441)	14.85 (268)	1.83 (33)	1805
1944/1965	4.19 (65)	80.28 (1246)	14.43 (224)	1.10 (17)	1552
1945/1966	3.73 (56)	81.24 (1221)	13.24 (199)	1.80 (27)	1503
1946/1967	3.64 (72)	79.93 (1581)	14.05 (278)	2.38 (47)	1978
1947/1968	2.90 (55)	77.37 (1470)	17.26 (328)	2.47 (47)	1900
1948/1969	3.69 (73)	79.25 (1612)	15.29 (311)	1.77 (36)	2034
1950/1970	2.97 (6)	77.23 (156)	18.81 (38)	0.99 (2)	202
1954/1974	3.67 (26)	78.53 (556)	15.82 (112)	1.98 (14)	708

## Appendix A

**Table A1:** Mean height, weight and BMI by year from1955 to 1974 (Source, AGMG).

Year of Birth/ Measurement	Height		Weight		BMI	
	*N*	Mean (SD)	*N*	Mean (SD)	*N*	Mean (SD)
1934/1955	2270	167.33 (6.45)	985	60.10 (8.91)	985	21.48 (2.46)
1935/1956	1993	167.50 (6.48)	1588	60.90 (8.41)	1588	21.78 (2.47)
1936/1957	2282	168.04 (6.64)	2194	61.31 (8.53)	2194	21.64 (2.46)
1937/1958	2129	167.42 (6.43)	1974	61.45 (8.25)	1973	21.90 (2.45)
1938/1959	2531	167.78 (6.56)	1955	61.98 (8.34)	1952	21.96 (2.53)
1939/1960	2067	168.15 (6.66)	1611	62.92 (8.94)	1611	22.11 (2.63)
1940/1961	2488	167.61 (6.62)	1527	63.45 (9.4)	1527	22.29 (2.67)
1941/1962	1608	169.13 (6.76)	1275	63.89 (8.98)	1275	22.21 (2.78)
1942/1963	2188	168.86 (6.66)	1884	64.25 (9.29)	1882	22.37 (2.74)
1943/1964	2105	168.88 (6.53)	1805	64.53 (9.38)	1805	22.53 (2.75)
1944/1965	2302	169.41(6.63)	1552	63.85 (8.96)	1552	22.32 (2.64)
1945/1966	1520	168.80 (6.55)	1505	64.07 (8.79)	1503	22.43 (2.81)
1946/1967	1999	169.43 (6.93)	1981	64.93 (9.32)	1978	22.57 (2.97)
1947/1968	2038	169.70 (6.73)	1901	66.10 (9.79)	1900	22.85 (2.94)
1948/1969	2062	170.03(6.69)	2036	65.47 (9.33)	2034	22.59 (2.80)
1950/1970	400	170.42 (6.97)	202	64.54 (10.51)	202	22.39 (2.98)
1954/1974	710	172.00 (6.68)	708	66.51 (9.57)	708	22.43 (3.00)

## Appendix B

**Table A2:** Nutrient intake per person and day in the city of Madrid, compared to the average for Spain and urban Spain, 1970 (Found own elaboration with data form Varela et al., 1971 [91].

	Madrid (City)	Spain	Urban Spain
Calories	2819	3105	2981
Proteins (g)	87.01	85.14	83.74
Fat (g)	115.04	117.84	117.01
Calcium (mg)	660	609	619
Iron (mg)	12.72	13.14	12.91
Retinol (I.U)	1623	1161	1398
β-Carotene (I.U)	2215	1978	2352
Vitamin A (I.U)	4022	3330	3969
Thiamine (mg)	1.2	1.25	1.21
Ribofravine (mg)	1.33	1.15	1.21
Niacin (mg)	13.56	13.56	13.32
Tryptophan (mg)	994	938	937
